# Response to: the nearly complete TME quality conundrum

**DOI:** 10.1007/s10151-018-1758-5

**Published:** 2018-02-23

**Authors:** M. Pędziwiatr, J. Witowski, P. Major, P. Małczak, M. Mizera, A. Budzyński

**Affiliations:** 1 2nd Department of General Surgery, Jagiellonian University Medical College, Kraków, Poland; 2 Centre for Research, Training and Innovation in Surgery (CERTAIN Surgery), Kraków, Poland

Dear Sir,

We would like to thank Prof. Bergamaschi and his colleagues for their commentary [1] on our systematic review and meta-analysis “There is no difference in outcome between laparoscopic and open surgery for rectal cancer: a systematic review and meta-analysis on short- and long-term oncologic outcomes” [2]. First of all, we appreciate Prof. Bergamaschi’s commentary since his reputation in the colorectal surgery community is indisputable. However, there are some points raised in his letter that must be addressed and explained because we believe that they are not entirely correct.

We agree that there are no data from 1966 to 2005 included in our meta-analysis. The reason is simple: there were no studies published in that time frame that matched our inclusion criteria. As described in methodology section of the paper, we screened databases covering a period from January 1966 to October 2016. Similarly, a recent meta-analysis by Martinez-Perez et al. [3] searched for papers from 1995, yet included studies published from 2003 onward. A 2003 study by Araujo et al. [4] was not included because it did not report circumferential resection margin status, an inclusion criterion for our review.

We would like to point readers (and Prof Bergamaschi) to Table 1 of our paper, where data on mean tumor distance from the anal verge are, in fact, reported.

We did not provide data on completeness of mesorectal excision in 6 out of 11 studies, since that information was not provided by authors of included studies themselves. We performed a pooled analysis on data from five studies (all studies that reported this outcome) which did not reveal significant differences.

Our grouping of “nearly complete” with “complete” mesorectal excision cases, based on Nagtegaal’s classification, was different from the meta-analysis by Martinez-Perez et al. In Nagtegaal’s original publication [5], the authors stated: “In our analysis we combined ‘optimal surgery’ cases and cases with nearly complete mesorectum, because we did not find statistical differences between these groups.” In Martinez-Perez’s meta-analysis, “nearly complete” and “incomplete” were incorrectly grouped together, as pointed out in the invited commentary to his review [6]. We decided to follow Nagtegaal’s original publication to reduce bias, and it turned out that this indeed changed the results!

We also agree that we did not analyze the design of included studies or study sample calculations (which were not provided in all studies), and this might have some influence on the final meta-analysis. However, we believe this is of minor importance, especially since the risk of bias in included studies was considered low. In our understanding, our meta-analysis of well-conducted randomized controlled trials (RCTs) provides a high level of evidence on the similarity of total mesorectal excision.

Both laparoscopic and open approaches to rectal cancer have their drawbacks. There is one certainty: open surgery has many limitations that cannot be overcome, whereas minimally invasive access, thanks to its ongoing technological advancement, is still evolving. Further comparison through RCTs of open and laparoscopic surgery is not needed in our opinion, and this is also evidenced by reluctance of patients to enroll in ongoing trials comparing open with laparoscopic surgery [7, 8]. Instead, we should wait for the results of studies comparing different laparoscopic/minimally invasive techniques to decide the future direction of rectal cancer surgery. This may not be using the robot to augment minimal access pelvic dissection [9], but maybe using a transanal TME when the COLOR III trial has reported [10]. Laparoscopic surgery has the same long-term oncological outcomes as open surgery in rectal cancer (leaving aside the other advantages of laparoscopic surgery). The debate should now focus around which is the best laparoscopic technique to use.
